# Pesticide Exposure and Hypertensive Disorders During Pregnancy

**DOI:** 10.1289/ehp.0900672

**Published:** 2009-06-04

**Authors:** Tina M. Saldana, Olga Basso, Donna D. Baird, Jane A. Hoppin, Clarice R. Weinberg, Aaron Blair, Michael C.R. Alavanja, Dale P. Sandler

**Affiliations:** 1 Social and Scientific Systems, Inc., Durham, North Carolina, USA; 2 Epidemiology Branch and; 3 Biostatistics Branch, National Institute of Environmental Health Sciences, National Institutes of Health, Department of Health and Human Services, Research Triangle Park, North Carolina, USA; 4 Occupational and Environmental Epidemiology Branch, Division of Cancer Epidemiology and Genetics, National Cancer Institute, National Institutes of Health, Department of Health and Human Services, Bethesda, Maryland, USA

**Keywords:** pesticide exposure, preeclampsia, pregnancy-induced hypertension

## Abstract

**Background:**

Hypertensive disorders of pregnancy, including pregnancy-induced hypertension (PIH) and preeclampsia (PE), complicate 2–8% of pregnancies. Few studies have examined environmental risk factors in relation to these conditions.

**Objectives:**

Our goal was to examine whether pesticide exposure during pregnancy was associated with hypertensive disorders of pregnancy.

**Methods:**

We analyzed self-reported data from 11,274 wives of farmers enrolled in the Agricultural Health Study (AHS) between 1993 and 1997. Using logistic regression models, we estimated the adjusted odds ratios (AORs) for PIH and PE associated with pesticide-related activities during the first trimester of pregnancy.

**Results:**

First-trimester residential and agricultural activities with potential exposure to pesticides were associated with both PIH [residential AOR = 1.27; 95% confidence interval (CI), 1.02–1.60; agricultural AOR = 1.60; 95% CI, 1.05–2.45] and PE (residential AOR = 1.32; 95% CI, 1.02–1.70; agricultural AOR = 2.07; 95% CI, 1.34–3.21).

**Conclusions:**

Exposure to pesticides during pregnancy may increase the risk of hypertensive disorders of pregnancy. Laboratory research may provide insights into relationships between pesticide exposure and hypertensive diseases of pregnancy.

Hypertensive disorders of pregnancy complicate 2–8% of all pregnancies (Zamorski and Green 2001), and their incidence may be increasing in the United States (Wallis et al. 2008). They include both pregnancy-induced hypertension (PIH), defined as newly diagnosed (during pregnancy) hypertension, and preeclampsia (PE), which is characterized by newly diagnosed hypertension accompanied by proteinuria. PE is a major cause of maternal and perinatal morbidity and mortality (MacKay et al. 2001; Wagner 2004; Zamorski and Green 2001), and severe PIH has been reported to have similar adverse effects (Allen et al. 2004; Ananth et al. 1995). Little is known about the potential impact of environmental factors in the etiology of these disorders.

Few environmental risk factors for hypertensive disorders of pregnancy have been examined. However, several studies have shown an association between pesticides and hypertension in general (Anand et al. 1990; Board on Population Health and Public Health Practice and Institute of Medicine 2007; Gordon and Padnos 2000; Morton et al. 1975; Sandifer et al. 1972; Smith et al. 2001; Warnick and Carter 1972). We recently reported an association between pesticide exposure during pregnancy and gestational diabetes (Saldana et al. 2007). Because insulin resistance is a known risk factor for PE, in this study we sought to examine the relation between pesticide exposure during pregnancy and the development of PIH and PE.

## Materials and Methods

### The Agricultural Health Study

Between 1993 and 1997, licensed pesticide applicators and their spouses from Iowa and North Carolina enrolled in the Agricultural Health Study (AHS) by completing several questionnaires. The cohort consists of > 57,311 private and commercial applicators (mostly male) and 32,171 spouses (mostly female) of private applicators who were farmers. Eighty-four percent of licensed applicators from Iowa and North Carolina enrolled in the study. Sixty-one percent (19,587) of the female spouses completed a Female and Family Health (FFH) questionnaire, with 18,335 reporting at least one pregnancy. Data for this study were obtained at the time of enrollment from both the applicator and spouse questionnaires, as well as the FFH questionnaire (Agricultural Health Study 1993), which provided information on reproductive health, including pregnancies that occurred before enrollment in the AHS. Information on exposures during pregnancy and pregnancy complications was collected only for the most recent pregnancy.

Of the 18,335 female participants who reported at least one pregnancy, we excluded women whose most recent pregnancy had occurred more than 25 years before enrollment in the study (*n* = 5,272), whose age at the most recent pregnancy was missing (*n* = 677), or who were < 16 or > 49 (*n* = 17) years of age at the time of the pregnancy. We further excluded women whose most recent pregnancy ended in a miscarriage, induced abortion, or molar pregnancy or was an ectopic pregnancy (*n* = 724) and those who reported that their pregnancy did not reach 20 weeks of gestation or were missing pregnancy outcome data (*n* = 106). Women with missing data on PIH, PE, or gestational diabetes mellitus (GDM; *n* = 24) or on pesticide-related activities during pregnancy (*n* = 198) were also excluded. Because age at diagnosis of chronic hypertension (HTN) was gathered in broad categories (< 20, 20–39, 40–59, > 60 years), we excluded women who reported HTN before age 20, women with HTN diagnosed between 20 and 39 years of age and pregnancy after age 39, and women who reported HTN but were missing age of diagnosis (*n* = 43). After these exclusions, 11,274 women remained for analysis.

The AHS was approved by the institutional review boards of the National Institutes of Health and its contractors. Informed consent was obtained from all participants. Additional details of the study are provided elsewhere (Alavanja et al. 1996).

### Exposure measures

To examine pesticide use, we used self-reported information about pesticide-related activities during the first trimester of the most recent pregnancy. Exposures during the second and third trimesters were not ascertained. We defined four pesticide exposure categories by combining activities with similar potential for pesticide exposure. The resulting categories were *a*) no exposure, *b*) indirect exposure (planting, pruning, weeding, picking, or harvesting), *c*) residential exposure (applying pesticides to the garden or inside the house), and *d*) agricultural exposure (mixing, applying pesticides to crops, or repairing pesticide application equipment). Women who reported activities pertaining to more than one category were classified according to the category reflecting the highest exposure potential (Saldana et al. 2007).

### Outcome measures

Women were asked, “Did you have pregnancy-induced high blood pressure during this pregnancy?” and separately “Did you have preeclampsia (toxemia) during this pregnancy?” We defined two case groups *a priori* as women reporting PIH only (without PE) and those reporting PE, 70% of whom also reported PIH. Thus, we had two separate case groups and one control group (those without either PIH or PE).

### Data analysis

We estimated the odds ratios (ORs) for PIH and PE through polytomous logistic regression models, treating the four exposure categories as a class variable. We used women with no reported exposure as the reference category. We adjusted for known predictors of PIH or PE for which we have data [participant’s race, age at the time of pregnancy, parity, and body mass index (BMI)] at enrollment (BMI at the time of pregnancy was not available). Smoking was not a confounder and was not included in our models. All adjusted models included these variables as well as state of residence. Variables were categorized as shown in Table 1. We also performed exploratory stratified analyses to describe risk of PIH and PE among women with and without GDM. We used the AHS phase 1 release P1REL0310.01 data. All statistical analyses were done using SAS, version 9.1 (SAS 2002).

## Results

The proportions reporting PIH only and PE were 5.9% and 4.5%, respectively. As expected, women with PE were more likely to be primiparous (Table 1). Women in each of the two case groups were heavier and more likely than controls to also report having gestational diabetes. Compared with women with a BMI of 18.5–24.9 kg/m^2^, those with a BMI of 25.0–29.9 and > 30 had ORs for PIH of 1.58 [95% confidence interval (CI), 1.29–1.95] and 2.47 (95% CI, 1.99, 3.07), respectively. The corresponding ORs for PE were 1.57 (95% CI, 1.24–1.98) and 2.23 (95% CI, 1.74–2.86).

First-trimester pesticide exposure was associated with both PIH and PE (Table 2) [chi-square tests (3 degrees of freedom) of the overall predictive contribution of the pesticide variable for the two outcomes had *p*-values of < 0.05 and < 0.01, respectively]. We saw a small increased risk of PIH and PE among women who reported having participated in activities that involved residential exposures (applying pesticides to home or garden), and a larger increase was associated with activities that involved agricultural exposure (mixing or applying pesticides or repairing pesticide-related equipment). To minimize the impact of misclassification due to poor reporting of events associated with pregnancies that occurred many years before enrollment, we repeated the analysis restricting to pregnancies that occurred within the 12 years before enrollment. The adjusted odds ratios (AORs) of the restricted analysis for pesticide exposure in relation to PIH were 1.32 (95% CI, 0.97–1.79) for residential exposure and 1.82 (95% CI, 1.07–3.10) for agricultural exposure. The corresponding ORs for pesticide exposure in relation to PE were 1.37 (95% CI, 0.96–1.95) and 1.99 (95% CI, 1.09–3.63). To examine the potential misclassification with early onset of chronic HTN, we repeated the analysis excluding all women reporting HTN between 20 and 39 years of age (*n* = 394) and found little change in estimates. The AORs for pesticide exposure in relation to PIH were 1.23 (95% CI, 0.96–1.58) for residential exposure and 1.60 (95% CI, 1.01–2.53) for agricultural exposure. The corresponding ORs for pesticide exposure in relation to PE were 1.28 (95% CI, 0.97–1.69) and 2.19 (95% CI, 1.39–3.45).

Because we previously reported an association between pesticides and GDM, and because many women with hypertensive disorders also reported GDM (8% of those with PIH and 24% of those with PE), we looked separately at women with and without GDM (Table 3). The association between pesticides and PIH were unchanged when the analysis was limited to women without GDM, the subgroup with sufficient numbers for analysis. The results for PE were less clear, although the association between agricultural pesticides and PE appears to be stronger for women also reporting GDM.

## Discussion

Our results suggest that the risk of both PIH and PE was elevated among women who performed activities likely to have exposed them to pesticides during their first trimester of pregnancy. Although the association between pesticide activities and PIH was also clearly evident among women who did not report GDM, the association between agricultural pesticide exposure and PE was most pronounced among women who also reported GDM.

Few studies have examined the relation between pesticide exposure and hypertensive disorders of pregnancy. In an occupational study of naval personnel, Irwin et al. (1994) found no relation between work involving exposure to unspecified hazardous chemicals and gestational hypertension or PE. The exposure status was, however, determined by the job title, which could result in bias toward the null, as pointed out by the authors. No increased risk of PE associated with either occupational or residential exposure to pesticides during pregnancy was found in another study that involved predominantly Hispanic women and focused on cholinesterase-inhibiting organophosphate and carbamate pesticides (Willis et al. 1993). The authors noted that the levels of exposure may have been very low, and that there may have been a lack of recognition and reporting of pesticides used in the home among the participants.

Though pesticide effects on pregnancy-related hypertensive disorders have rarely been investigated, several studies of hypertension outside pregnancy suggest a possible relation between pesticides, or specific chemicals, and hypertensive disorders. Studies from the 1970s showed a higher prevalence of hypertension among nonpregnant workers exposed to pesticides (Morton et al. 1975; Sandifer et al. 1972; Warnick and Carter 1972). A recent report on Vietnam Veterans concluded there was suggestive evidence of an association between TCDD (2,3,7,8-tetrachlorodibenzo-*p*-dioxin) and hypertension (Board on Population Health and Public Health Practice and Institute of Medicine 2007). In addition, several animal studies examined the relation between various pesticides and hypertension or blood pressure. Recent studies of the organophosphate-based insecticide chlorpyrifos have found increased blood pressure in exposed rats (Gordon and Padnos 2000; Smith and Gordon 2005). A study of non-pregnant rabbits exposed to organochlorine pesticides hexachlorocyclohexane and endosulfan also found significant increases in blood pressure and heart rates (Anand et al. 1990). Because we do not have information about specific pesticides used by the women during pregnancy, we cannot examine exposure involving these specific candidate pesticides.

As expected, we also found an increased risk of PE among women with GDM (Feig et al. 2006; Solomon and Seely 2001; Wolf et al. 2002). Diabetes can cause endothelial dysfunction (Calles-Escandon and Cipolla 2001; Dandona et al. 2006), which may contribute to the pathophysiology of PE (Lyell et al. 2003; Roberts and Lain 2002; Salas 1999), as supported by studies showing that tight glycemic control reduces the risk of PE in women who had diabetes before becoming pregnant (Crowther et al. 2005; Fan et al. 2006; Yogev et al. 2004). Another study also showed that increasing glucose intolerance among nondiabetics is also associated with PE (Sermer et al. 1995).

Our previous report of an association between pesticide exposure and gestational diabetes (Saldana et al. 2007) did not examine comorbidity with PE. Although the increased risk of PE associated with agricultural pesticide exposure among women with gestational diabetes may be attributable to reporting errors or a chance finding due to the small number of exposed women, it is possible that there are real exposure effects. For example, there may be genetic factors influencing the response to pesticides that lead to the co-occurrence of these conditions in susceptible women. Similarly, the vascular changes associated with GDM may enhance susceptibility to risk factors for PE. Alternatively, pesticide exposure may lead to a more severe form of hyperglycemia, which in turn could result in more endothelial damage and, ultimately, PE. Research to investigate such possibilities would require studies that focus on biological mechanisms.

Our study has a number of limitations. The disease status was self-reported. Although an earlier study found only fair reliability for reporting of several pregnancy events, including PE (Ellison et al. 2000), a recent study found 80% sensitivity and 96% specificity for self-reported PE compared with medical records over 20 years from the index pregnancy (Diehl et al. 2008). Our PIH and PE cases may have included some with chronic HTN. Our data on age of chronic HTN diagnosis were too broad to clearly determine prepregnancy HTN for some women; however, data from the second National Health and Nutrition Examination Survey (NHANES II) show that most chronic HTN occurs after 40 years of age (Geronimus et al. 1991), whereas most mothers in our study were < 40 years of age during their most recent pregnancy. Even among women in NHANES II with HTN between 20 and 39 years of age, most were older than 35 years; only 13% of the studied pregnancies were to women ≥ 35 years of age (Geronimus et al. 1991). Further, when we excluded all women reporting HTN between 20 and 39 years of age our results were essentially unchanged.

Our data on exposure to pesticides were based on a series of questions about the woman’s activities during the first trimester of the pregnancy. Data on the remainder of pregnancy were not collected, nor were the women asked about specific pesticides during the pregnancy. However, the activities listed allowed us to group similar tasks to assess the intensity of pesticide use. It has been shown that farmers provide reliable information regarding their personal pesticide use (Blair et al. 2002; Hoppin et al. 2002), and we have no reason to believe that reporting of pesticide use activities would differ by disease status.

We found the expected relationships with BMI and parity, suggesting that the data are generally valid (Bhattacharya et al. 2007). We did not see the expected protective effect of smoking on PE (Conde-Agudelo et al. 2008), but the prevalence of smokers was low among our sample of farm women (10%). To address the potential for inaccurate recall because our data span > 25 years, we carried out an analysis restricted to pregnancies occurring at most 12 years before reporting, which retained 53% of the sample. Our results were essentially unchanged.

The lack of information on other potential confounders was also a limitation. For example, we were unable to evaluate physical activity or solvent exposure as potential confounders. Physical activity has been reported to be associated with reduced risk of PIH and solvent use has been associated with hypertension more broadly (Eskenazi et al. 1988; Irwin et al. 1994). Although prepregnancy BMI would have been preferable, all we had were data on BMI at time of enrollment. Because BMI is related to risk of pregnancy disorders, it was important to assess its impact, and although there will have been some misclassification, women who are overweight early are more likely to be overweight later. Current BMI data were also missing for a large number of women. We included “missing” as a category in the analysis for the results shown. However, we evaluated the effects of limited BMI data by first dropping BMI from the models altogether and then by restricting analysis to women with nonmissing data. In both instances, the estimates of pesticide exposure remained essentially unchanged.

In summary, our analysis suggested a possible increased risk of both PIH and PE among women engaging in activities with potential for pesticide exposure during the first trimester of pregnancy. Follow-up of this observation will require more focused laboratory research on relationships among pesticide exposure, hypertension, and glucose intolerance.

## Figures and Tables

**Table 1:** Selected characteristics [no. (%)] for women in the hypertensive disorders analysis (*n*= 11,274), AHS (1993–1997).

Characteristic	No PE, no PIH (*n* = 10,110)	PIH only (*n* = 660)	PE (*n* = 504)
Maternal age at end of pregnancy (years)			
16–24	1,372 (14)	91 (14)	58 (11)
25–29	4,033 (40)	230 (35)	216 (43)
30–34	3,365 (33)	237 (36)	161 (32)
35–49	1,340 (13)	102 (15)	69 (14)

Race			
White	9,786 (98)	630 (97)	484 (98)
Other	177 (2)	20 (3)	9 (2)
Missing^a^	147	10	11

Education			
< High school	225 (3)	16 (3)	9 (2)
High school	3,382 (37)	202 (34)	157 (34)
> High school	5,526 (60)	374 (63)	292 (64)
Missing	977	68	46

Parity^b^			
0	1,108 (11)	106 (16)	101 (20)
1	3,937 (39)	254 (39)	186 (37)
2	3,072 (30)	179 (27)	140 (28)
≥ 3	1,993 (20)	121 (18)	77 (15)

BMI (kg/m^2^) at enrollment			
< 18.5	186 (2)	9 (2)	5 (1)
18.5–24.9	4,432 (52)	202 (36)	157 (38)
25.0–29.9	2,522 (29)	182 (33)	140 (34)
≥ 30	1,420 (17)	160 (29)	112 (27)
Missing	1,550	107	90

GDM			
No	9,775 (97)	608 (92)	385 (76)
Yes	335 (3)	52 (8)	119 (24)

Smoked during pregnancy			
No	9,055 (90)	594 (90)	444 (89)
Yes	1,012 (10)	65 (10)	58 (11)
Missing	43	1	2

State of residence			
North Carolina	2,488 (25)	229 (35)	134 (27)
Iowa	7,622 (75)	431 (65)	370 (73)

a Missing combined with other category for models.

b Parity (includes live births plus stillbirths before the index pregnancy).

**Table 2:** Risk of PIH and PE associated with pesticide exposure during pregnancy among wives of farmers in the AHS (1993–1997).

	No PE, no PIH		PIH only					PE				
Pesticide use	*n* = 10,110	(%)	*n* = 660	(%)	Crude OR	AOR^a^	95% CI	*n* = 504	(%)	Crude OR	AOR^a^	95% CI
None^b^	4,659	46	275	42	1.00	1.00	Reference	212	42	1.00	1.00	Reference
Indirect^c^	3,477	34	239	36	1.17	1.20	1.00–1.44	172	34	1.09	1.13	0.92–1.39
Residential^d^	1,689	17	120	18	1.20	1.27	1.02–1.60	95	19	1.24	1.32	1.02–1.70
Agricultural^e^	285	3	26	4	1.55	1.60	1.05–2.45	25	5	1.93	2.07	1.34–3.21

a Models adjusted for BMI at enrollment, mother’s age at pregnancy, parity, race, and state (variables categorized as shown in Table 1).

b Reference category.

c Includes planting, pruning, weeding, or harvesting.

d Includes applying pesticides to garden or inside house.

e Includes mixing or applying pesticides to crops or repairing pesticide application equipment.

**Table 3:** Risk of PIH and PE associated with pesticide exposure during pregnancy, among wives of farmers in the AHS (1993–1997), stratified by GDM.

	PIH only				PE			
	With GDM		Without GDM		With GDM		Without GDM	
Pesticide use	n1:n2^a^	AOR^b^ (95% CI)	n1:n2^a^	AOR^b^ (95% CI)	n1:n2 a	AOR^b^ (95% CI)	n1:n2^a^	AOR^b^ (95% CI)
None^c^	19:162	1.0	256:4,497	1.0	53:162	1.0	159:4,497	1.0
Indirect^d^	19:104	1.65 (0.80–3.37)	220:3,373	1.18 (0.98–1.43)	34:104	1.22 (0.72–2.05)	138:3,373	1.20 (0.95–1.52)
Residential^e^	12:55	1.60 (0.68–3.74)	108:1,634	1.23 (0.97–1.56)	16:55	1.00 (0.51–1.98)	79:1,634	1.47 (1.12–1.95)
Agricultural^f^	2:14	1.22 (0.25–6.04)	24:271	1.61 (1.04–2.50)	16:14	3.96 (1.72–9.09)	9:271	1.02 (0.51–2.02)

a n1 = number of cases in model; n2 = number of corresponding noncases in model.

b Models adjusted for BMI at enrollment, mother’s age at pregnancy, parity, race, and state (variables categorized as shown in Table 1).

c Reference category.

d Includes planting, pruning, weeding, or harvesting.

e Includes applying pesticides to garden or inside house.

f Includes mixing or applying pesticides to crops or repairing pesticide application equipment.
